# The speed acuity test as a diagnostic aid in cerebral visual impairment

**DOI:** 10.1038/s41598-022-14673-1

**Published:** 2022-06-22

**Authors:** Nouk Tanke, Annemiek D. Barsingerhorn, Jeroen Goossens, F. Nienke Boonstra

**Affiliations:** 1 Department of Cognitive Neuroscience, Donders Institute for Brain, Cognition and Behavior, Radboud University Medical Centre, Nijmegen, The Netherlands; 2 Department of Biophysics, Donders Institute for Brain, Cognition and Behavior, Radboud University, Nijmegen, The Netherlands; 3 Royal Dutch Visio, National Foundation for the Visually Impaired and Blind, Nijmegen, The Netherlands; 4 Behavioral Science Institute, Radboud University, Nijmegen, the Netherlands

**Keywords:** Visual system, Perception

## Abstract

One of the characteristics of children with cerebral visual impairments (CVI) is that they need more time to process visual information. However, currently, few tests are available that can reliably measure visual processing speed. The speed acuity test, a discrimination reaction-time test in which participants indicate the orientation of Landolt-C symbols as quickly and accurately as possible, was specifically developed to determine the time a child needs to discern visual details. The test measures both the accuracy and the latency of the responses for nine different optotype sizes in order to control for decreased visual acuity. The results show that children with CVI need significantly more time to respond to the largest optotype sizes than age-matched normally sighted children and children with visual impairments due to an ocular disorder (VI_o_). This effect is independent of the time it takes to make a motor response. However, the reaction-time difference between the children with CVI and VI_o_ is not seen for optotype sizes at the acuity threshold. Together with reaction times on visual and auditory detection tasks as controls, reaction times measured in the speed-acuity test allow for acceptable discrimination (AUC in ROC analysis: 0.81) between CVI and VI_o_.

## Introduction

Cerebral visual impairment (CVI) is a visual impairment that does not originate from an ocular deficiency, but is caused by malfunctions in the visual pathways of the brain^1^. The following definition is often used to describe CVI: all visual dysfunctions caused by damage to, or malfunctioning of, the retrochiasmatic pathways in the absence of any major ocular disease^2^. In children, CVI is most often caused by pre, peri or postnatal lack of blood or oxygen flow in the brain^2,3^. Examples of symptoms seen in children with CVI are difficulties in recognizing faces or objects, impaired perception of movement, hemianopsia, and difficulties seeing depth or contrast (for reviews, see^4,5^). In addition to this large variation in symptoms, children with CVI also display a large range in visual acuity and cerebral damage^6^. This variability makes CVI difficult to diagnose correctly, and almost impossible to diagnose with a single test. It is therefore important to have multiple tests available for each possible feature of CVI.

One symptom that is often suspected by parents and clinicians is that children with CVI need more time to process visual information. Unfortunately, there are few tests available that are specifically designed to measure visual processing speed. One way to test the time it takes for children to see visual information is by tracking the movement of the eyes. Using this method, it was found that children with CVI need significantly more time to move their eyes towards an image presented on screen than children from the age-matched control group and children with visual impairments due to ocular disorders (VI_o_)^7^. However, orienting to an image can be reflexive. Making an eye movement to a stimulus means that it has been detected and localized, but it does not imply that the child is able to discern the informational content of that stimulus. We therefore developed an inexpensive digital test that measures visual processing speed by determining the time it takes to recognize a visual stimulus presented on a computer screen^8^. In this test, children are asked to press the mouse button that corresponds to the location of the opening of a Landolt C. By showing stimuli of different sizes, the speed acuity test (SA) can measure the visual acuity of the child and his or her visual processing speed simultaneously. Preliminary data obtained with this SA-test in patients indicated that, as a group, children with CVI and children with VI_o_ need significantly more time to discern visual stimuli than normally sighted children (NS)^9^.

In the current study, we implemented the SA-test in a clinical setting at Royal Dutch Visio, a Dutch institute for visually impaired or blind people. Augmented data gathered during the implementation process was added to the former dataset^9^. The present paper not only confirms the former results in a larger group of children, the increased group sizes also allow us to extend the findings to differences between children with VI_o_ and children with CVI. Furthermore, this study describes the transition of the SA-test from a scientific test to a diagnostic aid for children with CVI.

## Results

**Figure 1:**
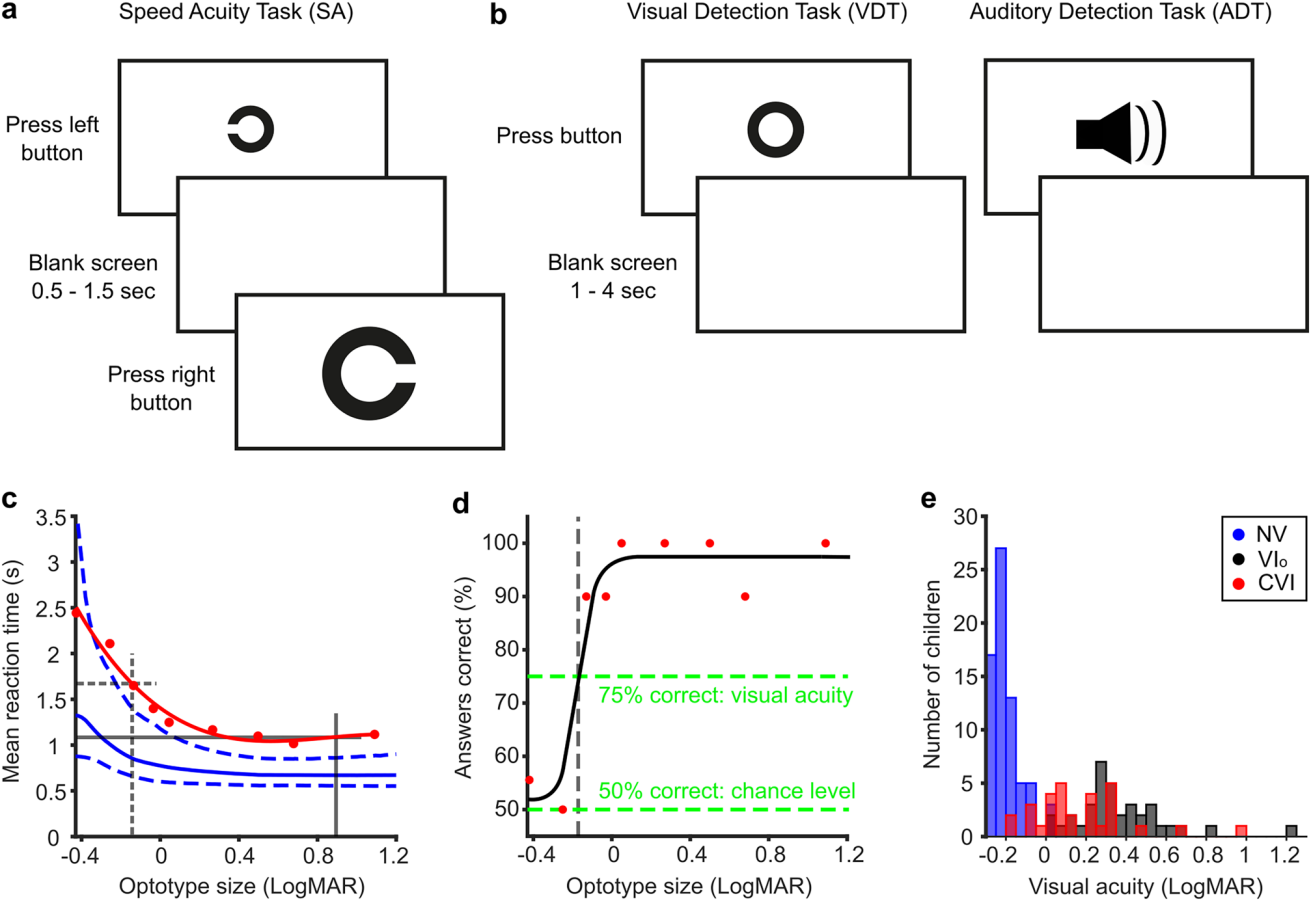
The speed acuity test (SA). Overview of the SA-test (**A**) in which children had to discriminate the orientation of the Landolt-C, and the detection tasks (**B**) in which children had to detect a large O-ring (VDT, left) or a sound (ADT, right) by pressing a button as soon as the stimulus appeared. (**C**,**D**) Chronometric and psychometric response functions obtained with the SA-test for one 9-year-old child having CVI with a normal visual acuity. (**C**) Subject’s mean reaction times for the different optotype sizes in red dots with the best fitting reaction time function. Dashed grey lines indicates mean reaction time at acuity-threshold, solid grey line indicates reaction time for large optotype sizes. In blue, the mean (solid line) chronometric response function of age-matched controls with normal vision and the corresponding 90% prediction interval indicated by the 5th and 95th percentiles (dashed lines) of the reaction-time distributions. (**D**) Subject’s percentage correct answers to each optotype size (red dots). The solid black curve is the fit to the psychometric data. The dashed grey line indicates the visual acuity. (**E**) Histogram that displays the distribution of visual acuities measured with FrACT for children with NS (blue), VI_o_ (black), and CVI (red). Produced in MATLAB 2020b (http://orcid.org/www.mathworks.com) and Inkscape 1.1.1 (http://www.inkscape.org).

Children participated in the SA-test (Fig. 1A), a visual detection task (VDT) and an auditory detection task (ADT, Fig. 1B). During the tests, a button had to be pressed in response to a stimulus. Figure 1C,D show the SA data from one 9-year-old child with CVI. His mean reaction time to each optotype size is shown in red (Fig. 1C). For the majority of the optotype sizes, the child needed more time to respond to the stimulus compared to the mean response of age-matched children with NS shown in blue. The delay of this child could not be explained by a low visual acuity, as the acuity of this child is normal. The latter can be inferred from the psychometric response function in Fig. 1D, which shows that the visual acuity of this child was around − 0.1 logMAR. Indeed, the acuities measured with the SA-test correlated strongly with the acuities measured with the traditional acuity test (FrACT, entire population; r = 0.93, p < 0.001; see^8^ for more detailed comparison). Figure 1E shows the acuities measured with the FrACT. Notice that the visual acuity of children with CVI ranged from − 0.2 to 0.96 LogMAR and overlapped with the visual acuities of both the children with NS (max 0.1 LogMAR) and VI_o_ (min 0.1 LogMAR). On average, the children with VI_o_ had a poorer acuity that the children with CVI (difference = 0.21 LogMAR, 95% CI 0.09–0.32 LogMAR). For the remainder of our analysis, the visual acuities measured with the FrACT were used to assure an independent assessment of visual acuity (see Supplemental Table 1 for patient characteristics).

### Children with VI_o_ or CVI compared to the control group

**Figure 2:**
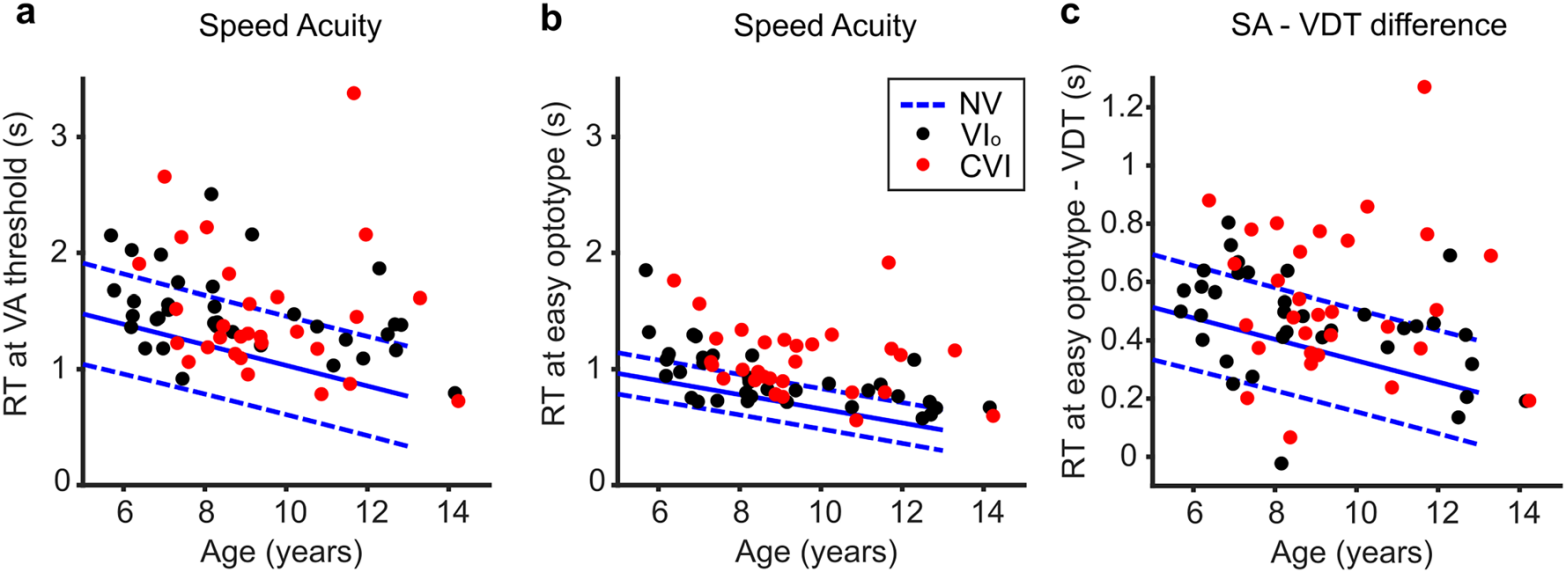
Mean reaction times in the speed acuity test. (**A**) Mean reaction times (RT) in the SA test at the acuity-threshold (as measured with the FrACT) plotted against age for children with VI_o_ (black dots) and children with CVI (red dots). In blue, the mean (solid line) reaction-time for NS children and corresponding 90% prediction interval indicated by 5th and 95th percentiles of the reaction-time distributions (dashed lines). (**B**) Same as (**A**) but for the mean reaction time (RT) to the two largest optotypes. (**C**) Same as (**A**) but for the mean reaction time (RT) to the easiest optotype during the SA-test minus the mean reaction time (RT) during the VDT. Produced in MATLAB 2020b (http://www.mathworks.com).

To compare SA responses between groups, the mean reaction times were calculated in response to the optotypes at the acuity-threshold (dashed grey lines in Fig. 1C) and the mean reaction times to the two biggest optotypes (solid grey lines in Fig. 1C). Figure 2 shows the mean reaction times per child plotted against age. Note that reaction times decrease with age, which is why age is added as a covariate in the regression analyses (see Supplementary Table 2 for a listing of the resulting regression coefficients). The NS children needed on average 1191 ms (95% CI 1090–1290 ms) to respond to the optotype sizes at the acuity-threshold and 715 ms (95% CI 678–751 ms) for the largest optotype sizes. Children with reaction-times exceeding the 95th percentile of age-matched controls were counted as being slow. While 13/36 (36%) of the children with VI_o_ scored above the 95th percentile of age-matched controls for the reaction times at the acuity-threshold, 20/36 (56%) did so for the large optotype sizes. The two proportions are not significantly different (Odds ratio 0.45, p = 0.15, Fisher’s exact test). For the children with CVI, 12/30 (40%) scored above the 95th percentile of age-matched controls for the reaction times at the acuity-threshold, compared to 24/30 (80%) for the large optotype sizes; which is a significantly higher proportion of children (Odds ratio 0.17, p = 0.003, Fisher’s exact test). On average, the children with VI_o_ and CVI needed significantly more time than the age-matched controls on both the small optotypes (Fig. 2A, VI_o_; 264 ms longer (95% CI 70–459 ms), t(159) = 2.68, p = 0.008, CVI; 412 ms longer (95% CI 204–620 ms), t(159) = 3.91, p < 0.001, linear regression) and the large optotype sizes (Fig. 2B, VI_o_; 183 ms longer (95% CI 113–254 ms), t(159) = 5.13, p < 0.001, CVI; 367 ms longer (95% CI 291–442 ms), t(159) = 9.60, p < 0.001, linear regression).

One factor that can cause delayed SA responses is the child’s motor ability (a button needs to be pressed). The difference between the response times in the SA test and the response time to the VDT filters out the motor response as well as other delays that remain constant, such as sensory afferent delays and cognitive delays that are unrelated to the visual discrimination process. What is left is the time it takes to make the choice between leftward and rightward orientation of the Landolt C. Both children with CVI and VI_o_ needed significantly more time to detect the stimulus in the VDT than NS age-matched controls (data not shown, VI_o_; 103 ms longer (95% CI 58–147 ms), t(159) = 4.57, p < 0.001, CVI; 187 ms longer (95% CI 140–235 ms), t(159) = 7.81, p < 0.001, linear regression). The time difference between discriminating the largest optotypes in the SA test and detecting the stimulus in the VDT was, on average, 365 ms (95% CI 332–397 ms) for NS children. For children with CVI, 13/30 (43%) children scored above the 95th percentile for NS children, and their mean SA-VDT reaction-time difference was significantly higher than that of the NS group (Fig. 2C, 179 ms higher (95% CI 112–246 ms), t(159) = 5.29, p < 0.001, linear regression). For children with VI_o_, the average difference with the control group was small, but significant (Fig. 2C, 81 ms higher for VI_o_ (95% CI 18–143 ms), t(159) = 2.54, p = 0.012, linear regression); only 9/36 (25%) children with VI_o_ scored above the 95th percentile for NS children.

Besides affecting a child’s motor abilities and visual functions, cerebral impairments may also affect the processing of other types of sensory information. NS children needed, on average, 290 ms (data not shown, 95% CI 256–325 ms) to respond to the ADT, which is significantly less than for the VDT (350 ms, 95% CI 327–373 ms). Surprisingly, we found that not only children with CVI, but also children with VI_o_, needed significantly more time to respond to the sound in the ADT than NS children (data not shown, VI_o_; 112 ms longer (95% CI 44–179 ms), t(156) = 3.27, p = 0.0013, CVI; 246 ms longer (95% CI 176–317 ms), t(156) = 6.89, p < 0.001, linear regression).

**Figure 3:**
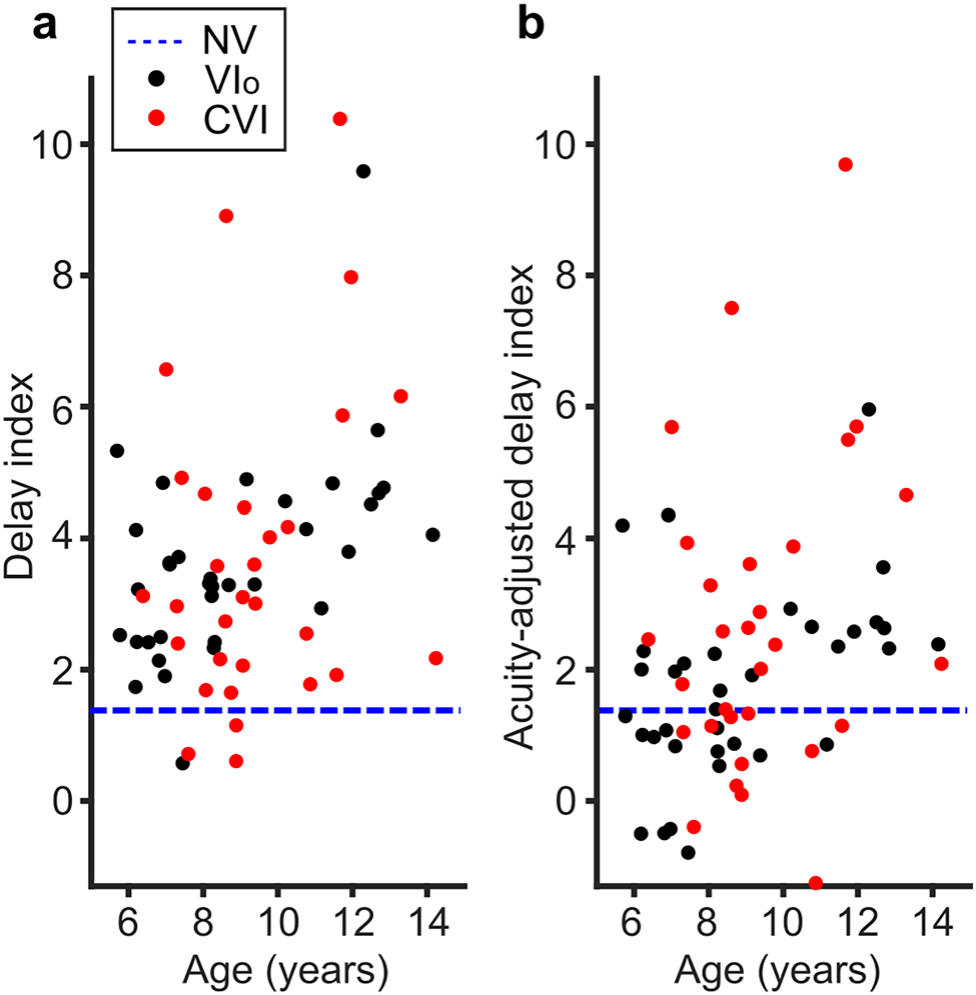
Delay index. (**A**) The delay indices for children with VI_o_ (black dots) and children with CVI (red dots) plotted against age. In blue, 95th percentile (dashed lines) of children with NS. (**B**) Same as (**A**), but for the acuity-adjusted delay indices which take into account the subjects’ reduced visual acuity as measured with the SA-test. Produced in MATLAB 2020b (http://www.mathworks.com).

To find out whether the delayed SA score holds when we take into account the entire chronometric curve, we used a delay index (DI, Fig. 3A). The DI compares the reaction times to all optotype sizes to the mean age-matched control data, measured in standard deviations (sd). For example, when a child has a DI of 2, this indicates that their reaction time is on average 2 standard deviations above the average performance of NS children of the same age. Most of the children with VI_o_ (35/36: 97%) and CVI (27/30: 90%) had a DI larger than the 95th percentile of the NS children. The mean DI was 3.8 sd (95% CI 3.2–4.4 sd) for children with VI_o_ and 3.7 sd (95% CI 2.8–4.6 sd) for children with CVI, which was significantly higher than the average DI of 0.3 sd (95% CI − 0.1 to 0.6 sd) of NS children (VI_o_; 3.5 sd higher (95% CI 2.9–4.1 sd), t(160) = 11.22, p < 0.001, CVI; 3.4 sd higher (95% CI 2.8–4.1 sd), t(160) = 10.42, p < 0.001, linear regression).

To test if the longer reaction times were related to a reduced visual acuity, we calculated acuity-adjusted DIs (Fig. 3B). An acuity-adjusted DI of zero is expected if reduced visual acuity alone accounts for the delayed response. The average acuity-adjusted DI was indeed lower than the regular DI for both the children with VI_o_ (1.9 sd, 95% CI 1.4–2.4 sd) and CVI (2.7 sd, 95% CI 1.8–3.5 sd), The majority of the children with VI_o_ (20/36: 55.6%) and CVI (19/30: 63.3%) still had a DI higher than the 95th percentile of the control group, and the DIs of both groups remained, on average, significantly higher than the NS children (VI_o_; 2.7 sd higher (95% CI 2.1–3.3 sd), t(160) = 8.75, p < 0.0001, CVI; 3.5 sd higher (95% CI 2.8–4.1 sd), t(160) = 10.47, p < 0.001, linear regression). These results confirm that the previously documented effects^9^ remain similar with a more extensive dataset. The previous dataset however, was too small to draw conclusions about differences between the VI_o_ and CVI children.

### Differences in SA scores between children with VI_o_ and children with CVI

The current study shows that there is no difference in reaction times to the threshold stimuli between the children with VI_o_ and CVI (Fig. 2A, 148 ms longer for CVI (95% CI − 98 to 394 ms), t(159) = 1.19, p = 0.24, linear regression). However, the children with CVI did need more time than the children with VI_o_ to discriminate the largest optotype sizes (Fig. 2B, 183 ms longer for CVI (95% CI 94–273 ms), t(159) = 4.05, p < 0.001, linear regression), also when the SA score was adjusted for the VDT time (Fig. 2C, 99 ms longer for CVI (95% CI 19–178 ms), t(159) = 2.46, p < 0.02, linear regression), even though VDT times were already 85 ms longer (95% CI 28–141 ms) in children with CVI compared to children with VI_o_. The reaction times in the ADT were also longer for children with CVI than children with VI_o_ (135 ms longer for CVI (95% CI 50–219 ms), t(159) = 3.15, p < 0.005). Surprisingly, no significant differences were found between the two groups for the DI (Fig. 3A, − 0.1 sd lower for CVI (95% CI − 0.89 to 0.69 sd), t(160) = − 0.25, p = 0.81, linear regression). The mean difference between the children with VI_o_ and CVI increased when the DI was adjusted for visual acuity, however, the difference remained non-significant (Fig. 3B, 0.77 sd higher for CVI (95% CI − 0.01 to 1.54 sd), t(160) = 1.94, p = 0.054, linear regression). For a summary of these results, see Supplementary Table 3.

### Classification

**Figure 4:**
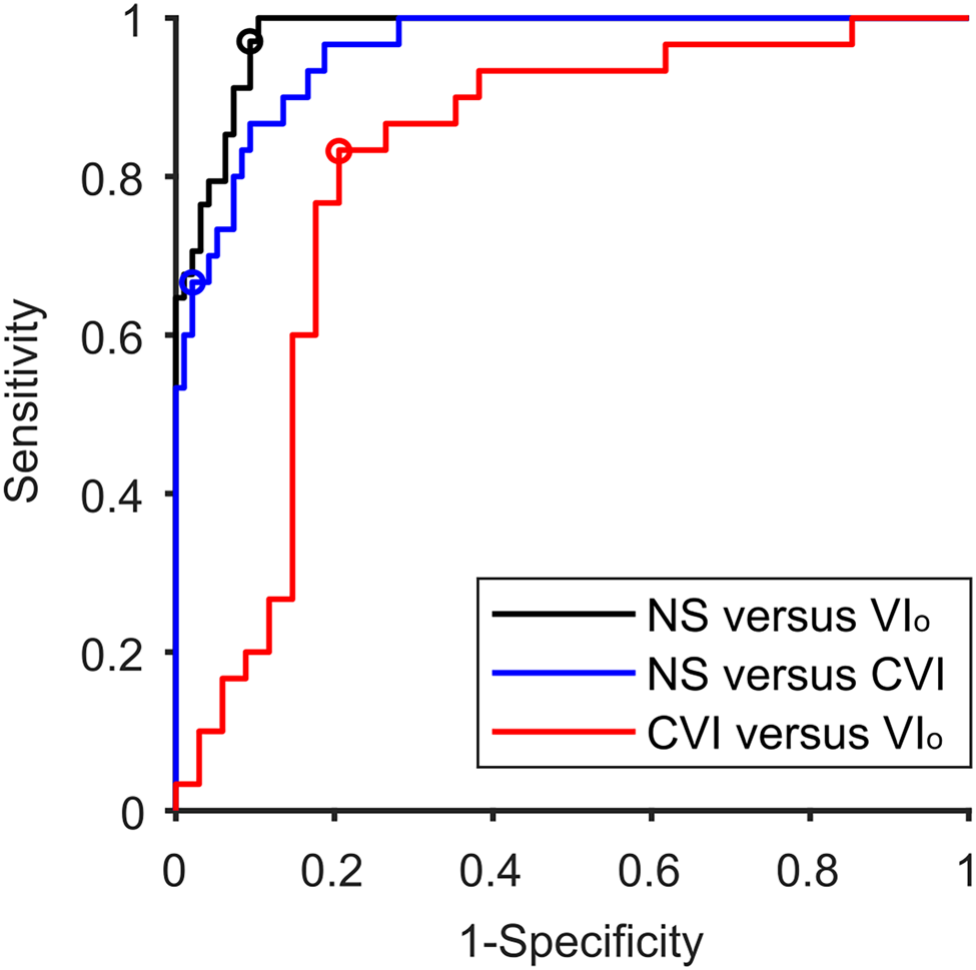
ROC analysis. Sensitivity and specificity of two logistic regression classifiers that distinguished NS children from children with VI_o_ (black) or CVI (blue) and a third logistic regression classifier that distinguished children with VI_o_ from children with CVI (red). Circles indicate the optimal point for each classifier. Produced in MATLAB 2020b (http://www.mathworks.com).

Figure 4 shows the classification performance of three logistic-regression classifiers. The first two distinguished either between NS and VI_o_ (black) or between NS and CVI (blue), while the third discriminated between VI_o_ and CVI (red). The classifiers only used reaction time data from the SA-test, augmented with VDT and ADT scores (see “Methods”). Visual acuity was not included. The ROC curve for the first classifier (NS versus VI_o_) had an AUC value of 0.98 (95% CI 0.95–0.99) with a sensitivity and specificity at the optimal point of 0.97 and 0.91, respectively. The second classifier (NS versus CVI) had an AUC value of 0.96 (95% CI 0.91–0.98), with a sensitivity and specificity at the optimal point of 0.67 and 0.98, respectively. This indicated that the combination of reaction time scores from the SA, VDT and ADT allowed for excellent discrimination between children NV and children with VI_o_ or CVI. The discriminability between children with VI_o_ and children with CVI was not as good, but with an AUC value of 0.81 (95% CI 0.66–0.90), the performance of this classifier is still acceptable. Its sensitivity and specificity at the optimal point was 0.83 and 0.79, respectively.

## Discussion

This study shows that children with VI_o_ and children with CVI need significantly more time to recognize where the opening of a Landolt C is located than age-matched controls. These effects are independent of motor (dis)abilities and visual acuity. Differences between children with CVI and children with VI_o_ are likely to be of visual origin. Children with CVI need, on average, more time to respond to clearly discernible optotypes than children with VI_o_. However, no difference is found between the two groups in the reaction time to an optotype size at the acuity-threshold. Our results thus indicate that the difference between children with CVI and those with VI_o_ in visual processing speed is measured predominantly in their response time to easy stimuli. We therefore believe the SA-test to be a useful addition to the diagnostic test battery to distinguish CVI from VI_o_ and NS.

Children with VI_o_ and children with CVI show longer visual discrimination times compared to age-matched controls. Additionally, children with CVI need more time to detect visual information than children with VI_o_. However, thirteen children from the patient groups (CVI, n = 11, VI_o_; n = 2) had some degree of motor impairment, which could also introduce delays in their responses. Subtracting the VDT reaction time from the SA time filters out the time needed to make a motor response. Even when the SA time is corrected for the time it takes for the children to perform the motor response, the children with CVI were slower than children with VI_o_. The longer discrimination times in CVI thus reflect additional impairment in visual decision-making in CVI.

Note that 11 out of 30 children with CVI showed some degree of cognitive disfunction (Supplementary Table 1). Additionally, both the children with CVI and the children with VI_o_ needed more time to respond to auditory information than the NS children. This indicates that both patient groups show, on average, a delay in their ability to process sensory information in general. It is therefore possible that cognitive or sensory factors other than visual processing speed affect SA performance. However, such factors are unlikely to change with optotype size. By measuring responses to different optotype sizes, the SA-test allows us to zoom in on problems that are most likely of visual origin.

Interestingly, we found evidence that shape of the chronometric response functions is indeed different between children with CVI and those with VI_o_; the mean reaction times to the optotype size at the acuity-threshold show no significant difference between children with VI_o_ and children with CVI but the mean reaction times for the largest optotypes did. This suggests that when the stimuli are hard to see, both groups perform similarly, but when the stimuli become easier, children with VI_o_ are less limited by their visual impairment. It indicates that for children with VI_o,_ who have no cerebral problems, magnification helps to compensate for their visual impairment in terms of visual processing speed. Whereas for children with CVI, it is likely that magnification cannot help to overcome brain damage, and that, in general, they will need more time to process visual information. This would also explain why, when the entire response curve is compared between groups, there is no significant difference between the two patient groups even when the curves are adjusted for acuity.

Possibly, our findings underestimate the differences between children with VI_o_ and children with CVI because the visual acuities of the children in the CVI group were on average better than those of children with VI_o_. Perhaps, the distinction would have been clearer if the group of children with CVI included a broader range of acuities. Additionally, four children with CVI were excluded from the SA-test because their response accuracy to the biggest optotypes was deemed too low. This did not occur in the VI_o_ group or any of the NS children. This too indicates that the differences between CVI and VI_o_ might be larger than reflected in our study. In general, children with severe cognitive and/or motor disabilities might not be capable to perform the SA-test, thereby limiting the CVI-population that can be assessed with this test.

Together with the VDT and ADT, the SA test results offer an acceptable level of sensitivity and specificity in discriminating between children with VI and CVI. It is important to note, however, that there is substantial overlap in performance between the two patient groups. It is not recommended, therefore, to use these tests as a sole diagnostic criterion. Even so, we do believe the SA-test to be a valuable addition in the clinical setting when a general delay in visual processing time is suspected. Because the SA-test is an easy-to-use test that can accurately measure visual processing speed as well as visual acuity, it can help us understand problems in visually guided behaviors in a child’s daily life. It is suitable for children with a (developmental) age of 5 years and up and, with the big buttons, it can be used for children who have physical disabilities and or a delay in motor development. Children with CVI who used the big buttons needed more time to respond during the SA-test and VDT than children who used the computer mouse (Supplementary Fig. 1). However, the difference between computer mouse and big buttons was not seen in the children with VI_o_. It is therefore likely that the discrepancy between the two datasets was not caused by the type of buttons used, but by the inclusion of children with more severe symptoms.

In conclusion, by comparing reaction times in a Landolt C orientation discrimination task with reaction times in a visual or auditory detection task, it is possible to measure the time it takes to recognize visual information, independent of visual acuity or motor functioning. By measuring chronometric functions, the SA-test can help to zoom in on problems in visual processing and visually guided decision-making, and it can accurately detect a reduction in visual processing speed in children with CVI compared to age-matched children with normal vision and with VI_o_. The test has been implemented in a clinical setting and proves a fitting addition to the current array of diagnostic tests for CVI.

## Methods

### Participants

A total number of 166 children aged 5–16 years were recruited. All the children had a binocular visual acuity better than 1.3 LogMAR. For the NS children (n = 97, 9.3 ± 2.0 (mean ± sd) years) and children with VI_o_ (n = 36, 9.0 ± 2.8 (mean ± sd) years), inclusion criteria were; normal birth weight (> 2500 g), birth at term (> 36 weeks), no perinatal complications and normal development. NS children had a linear distant visual acuity of 0.1 logMAR or better. Children with VI_o_ had a visual acuity worse than 0.1 LogMAR. The only inclusion criterium for the children with CVI (n = 34, 9.4 ± 1.9 (mean ± sd) years) was having the diagnosis of CVI. The diagnosis of CVI was made by ophthalmologists of Bartiméus or Royal Dutch Visio, Dutch institutes for the rehabilitation of the visually impaired. After 2019, the Dutch CVI guidelines were officially applied to obtain the diagnosis^10^. Children with glasses wore them during all tests. NS children were recruited from three different primary schools and testing occurred at these locations. Children with VI_o_ and CVI were recruited from Bartiméus (used computer mouse, VI_o_; n = 29, CVI; n = 17) or Royal Dutch Visio (used big buttons, VI_o_; n = 7, CVI; n = 17) and tested at these institutes. For details concerning the children’s diagnoses, see Supplementary Table 1.

Informed consent was obtained from the parents of all participants. The study was approved by the local ethics committee Commissie Mensgebonden Onderzoek regio Arnhem-Nijmegen, The Netherlands (protocol NL48708.091.14), and conducted according to the principles of the Declaration of Helsinki.

For detailed descriptions of the methods used during this study, see^8,9^. Here, we duplicate part of those methods’ descriptions for clarity.

### The speed acuity test (SA)

The SA-test was administered binocularly at 5 m. Each trial consisted of a high-contrast black Landolt-C presented at the center of the computer screen against a white background. Children had to indicate, as quickly and accurately as possible, on which side, right or left, the opening of the C was located by pressing the corresponding mouse button (Fig. 1A). For the set-up at the Royal Dutch Visio, two big buttons (Supplementary Fig. 1A, ø 125 mm each, one blue, one yellow) were used that were equivalent to mouse buttons (Eelke Verschuur, Smoothie groot). The stimulus was presented until the child responded after which a blank screen appeared for 0.5–1.5 s. Task difficulty was manipulated by presenting nine optotype sizes (− 0.43, − 0.25, − 0.13, − 0.03, 0.05, 0.27, 0.5, 0.68 and 1.09 LogMAR for children with a visual acuity of 0.1 LogMAR or better), each presented 10 times in pseudo random order. Optotype sizes were adjusted to the child’s visual acuity before the start of the SA-test (acuity between 0.1 and 0.5 LogMAR; optotype sizes − 0.13, − 0.03, 0.05, 0.27, 0.5, 0.68, 0.8, 1.09 and 1.3 LogMAR, acuity of 0.5 LogMAR or worse; optotype sizes − 0.03, 0.05, 0.27, 0.5, 0.68, 0.8, 1.09, 1.3 and 1.5 LogMAR).

### Visual detection task (VDT) and auditory detection task (ADT)

The children also performed a visual detection task (Fig. 1B) at ~ 65 cm distance to measure the time children needed to respond to a supra-threshold stimulus. In the visual detection task (20 trials), they had to press a mouse button (or a big button at Royal Dutch Visio) as soon as they saw the visual stimulus (a large high-contrast black letter ‘‘O’’). Similar to the VDT, the children participated in the ADT where a sound was played instead of a visual stimulus. For details, see^8^.

### Equipment

The stimulus software used at the schools and Bartiméus was written in Matlab (version 2013b) using the Psychophysics Toolbox (version 3.0.12^11^). At Royal Dutch Visio, the stimulus software was written in Python using PsychoPy3 (version 2020.2.10^12^). The visual stimuli were presented on a 23-inch LCD screen (Dell, Inc. 1920 × 1200 pixels).

### Procedure

To assess the visual acuity binocularly, children first participated in the Freiburg visual acuity test (FrACT^13^) administered at 5 m distance. Secondly, the SA-test was administered at the same distance. Finally, the computer screen was moved to 65 cm distance for the visual and auditory detection tasks. The distance was different between SA and the detection tasks because the detection tasks were part of an extended test battery that required reading-distance.

### Data analyses

The offline analysis was performed and images were created using Matlab (version 2020b).

#### SA, VDT and ADT

Mean reaction times were computed after removing atypically long or short reaction times. Trials were excluded if the reaction time deviated more than three times the median absolute deviation from the median after discarding reaction times < 0.1 s. Data from the SA-test were excluded from further analysis when the median of their accuracy scores for the 5 largest optotypes was lower than 87.5% correct, because it indicated poor compliance with the task (n = 4/34 CVI children aged 5.2, 7.0, 7.1 and 8.1 years). The SA-visual acuity was determined from a cumulative Gaussian function fitted to the accuracy data, at the point where the curve crossed the 75% threshold (i.e., half way of 100% correct performance and pure guessing in a two alternative forced choice paradigm).

SA time at VA threshold was determined from the chronometric response functions fitted to the reaction time data (see Fig. 1C for illustration and^8^), or if this fit was unreliable, as the mean reaction time to the two optotype sizes that were closest in size to the child’s visual acuity. SA time for large optotype sizes was determined as the mean reaction time to the two largest SA optotype sizes. SA-VDT was determined as the mean reaction time to the largest two SA optotype minus the mean reaction time to the VDT.

#### Delay index

The mean of the differences in response time between the child’s reaction time and the age-matched norm value at each optotype size. For the acuity-adjusted DI, the reaction times curves of age-matched controls were shifted to the right (towards larger optotype sizes) based on the child’s visual acuity. For equations and more detailed descriptions of the DI, see^9^.

#### Linear regression

Differences between the three groups (NV, VI_o_ and CVI) were evaluated with analysis of covariance using a multiple linear regression model that includes age as covariate (model: *Test_Result* ~ *group* + *age,* Wilkinson notation). In these analyses, age was centred around the mean age of the children with NV.

#### Classification by logistic regression

We used logistic regression models with four predictors to distinguish between the children’s conditions. The four predictors included were; the SA times to large optotypes, the VDT and ADT reaction times, and the DI index. The SA, VDT and ADT times were adjusted for age. The first two classifiers distinguished between NS and VI or CVI, while the third classifier distinguished between VI and CVI. Classification performance was evaluated with Receiver Operating Characteristic (ROC) curves. We used bootstrapping (n = 10,000) to estimate confidence intervals for the Area Under the Curve (AUC) values.

## Supplementary Information

Supplementary Information.

## Data Availability

The raw data supporting the conclusions of this article will be made available by the authors upon request.
