# Supplementary material for: The speed acuity test as a diagnostic aid in cerebral visual impairment
Source: Sci Rep. 2022 Jun 22;12:10596. doi: 10.1038/s41598-022-14673-1 (PMC9217927; doi:10.1038/s41598-022-14673-1)
Supplement: Supplementary file 1 — Supplementary Information. [file 41598_2022_14673_MOESM1_ESM.pdf]

The speed acuity test as a diagnostic aid in cerebral visual impairment

Nouk Tanke[1], Annemiek D. Barsingerhorn[1,2], Jeroen Goossens[1], F. Nienke Boonstra[1,3,4*]

[1]Department of Cognitive Neuroscience, Donders Institute for Brain, Cognition and Behavior, Radboud University Medical Centre, Nijmegen, the Netherlands
[2]Department of Biophysics, Donders Institute for Brain, Cognition and Behavior, Radboud University, Nijmegen, the Netherlands
[3]Royal Dutch Visio, National Foundation for the Visually Impaired and Blind, Nijmegen, the Netherlands.
[4]Behavioral Science Institute, Radboud University, Nijmegen, the Netherlands

Supplementary information

Supplementary Table 1. Clinical characteristics of the children with VI_o and CVI

Participant number (Pp), age, visual acuity in LogMAR (VA), group, diagnosis, and the presence of motor impairment, mental delay, strabismus and nystagmus (+: manifest, +/-: latent, -: absent).

Pp	Age	VA	Group	Diagnosis	Motor imp.	Mental delay	Strabismus	Nystagmus
**1**	8	0.3	VI_o	Optic nerve hypoplasia	-	-	+/-	-
**2**	5	0.1	VI_o	Hypermetropia	+	-	-	-
**3**	5	0.4	VI_o	Incontinentia pigmenti	-	-	+	+
**4**	6	0.3	VI_o	Congenital stationary night blindness (CSNB)	-	-	-	-
**5**	8	0.3	VI_o	Infantile nystagmus syndrome	-	-	-	+
**6**	9	0.1	VI_o	Hypermetropia	-	-	+	-
**7**	6	0.3	VI_o	Albinism	-	-	-	+/-
**8**	8	0.2	VI_o	Macular hypoplasia	-	-	+	+
**9**	8	0.3	VI_o	Infantile nystagmus syndrome	-	-	+	+
**10**	12	1.2	VI_o	Congenital stationary night blindness (CSNB)	-	-	-	+
**11**	12	0.3	VI_o	Congenital stationary night blindness (CSNB)	-	-	-	-
**12**	6	0.5	VI_o	Albinism	-	-	+/-	+
**13**	6	0.3	VI_o	Congenital stationary night blindness (CSNB)	-	-	-	+
**14**	12	0.4	VI_o	Infantile nystagmus syndrome	-	-	-	+
**15**	7	0.1	VI_o	Infantile nystagmus syndrome	-	-	-	+
**16**	6	0.6	VI_o	Albinism	-	-	+/-	+
**17**	11	0.2	VI_o	Macular atrophy	-	-	-	-
**18**	10	0.8	VI_o	Cone-rod dystrophy	-	-	-	-
**19**	6	0.1	VI_o	Hypermetropia	-	-	+	+
**20**	8	0.1	VI_o	Albinism	-	-	+	-
**21**	7	0.3	VI_o	Albinism	-	-	+	+/-
**22**	11	0.5	VI_o	Albinism	-	-	-	+
**23**	7	0.4	VI_o	Cone dysfunction (Bornholm)	-	-	+/-	-
**24**	6	0.2	VI_o	Coloboma of the iris and retina, and optic nerve	-	-	+	-
**25**	10	0.4	VI_o	Albinism	-	-	+/-	+

**Pp**	**Age**	**VA**	**Group**	**Diagnosis**	**Motor imp.**	**Mental delay**	**Strabismus**	**Nystagmus**
**26**	9	0.6	VI$_o$	Aniridia	-	-	+	+
**27**	12	0.5	VI$_o$	Albinism	-	-	+	+
**28**	11	0.3	VI$_o$	Infantile nystagmus syndrome	-	-	-	+
**29**	7	0.7	VI$_o$	Congenital stationary night blindness (CSNB)	-	-	+	+
**30**	16	0.5	VI$_o$	Dominant cystoid macular dystrophy	-	-	-	-
**31**	12	0.3	VI$_o$	Congenital ptosis	+	-	-	-
**32**	8	0.2	VI$_o$	Hypermetropia	+/-	+	-	-
**33**	8	0.3	VI$_o$	Hypermetropia with astigmatism	-	-	-	-
**34**	14	0.4	VI$_o$	Infantile nystagmus syndrome	-	-	-	+
**35**	6	0.5	VI$_o$	Hypermetropia with astigmatism	-	-	-	+
**36**	6	0.3	VI$_o$	Hypermetropia	-	-	+	-
**37**	9	0.5	CVI	Noonan syndrome	-	-	+	+/-
**38**	9	0.3	CVI	Noonan syndrome	+	-	+/-	+/-
**39**	11	1.0	CVI	Status after meningitis and cerebritis	-	+	-	-
**40**	7	0.3	CVI	Premature	-	+	+	-
**41**	10	0.7	CVI	Premature	-	-	-	-
**42**	8	0.3	CVI	Optic nerve atrophy, microcephalus and bilateral occipital infarcts	-	-	+	+/-
**43**	7	0.1	CVI	Premature and dysmature	+	-	+	-
**44**	8	0	CVI	Joubert syndrome	-	-	+	-
**45**	9	-0.2	CVI	Cerebral arteriovenous malformation: 2 strokes	-	-	-	-
**46**	8	-0.2	CVI	Premature	-	+	-	-
**47**	11	0.3	CVI	White matter damage due to mitochondrial disease and internuclear ophthalmoplegia	+	-	+	+
**48**	8	-0.1	CVI	Premature and perinatal complications	+	-	+	-

Pp	Age	VA	Group	Diagnosis	Motor imp	Mental delay	Strabismus	Nystagmus
**49**	10	0	CVI	Premature	+	-	-	-
**50**	8	0.1	CVI	Perinatal complications	-	+	+	-
**51**	7	0.3	CVI	Dysmature, partial cataract	-	-	-	-
**52**	10	0.1	CVI	Exact cause unknown	+	+	-	-
**53**	7	-0.1	CVI	Exact cause unknown	+	-	-	-
**54**	11	0	CVI	High-energy trauma	-	-	-	-
**55**	8	0	CVI	Neurofibromatosis 1	-	-	-	-
**56**	8	0.2	CVI	Premature and perinatal complications	-	-	-	-
**57**	14	0	CVI	Cerebral palsy	+	+	-	-
**58**	11	0.2	CVI	High-energy trauma	-	-	-	+
**59**	8	0.2	CVI	Exact cause unknown	-	-	-	-
**60**	6	0.1	CVI	Unilateral heterotopia	+	-	-	-
**61**	9	0.1	CVI	KDM5C-syndrome	-	+	-	-
**62**	9	0.1	CVI	Perinatal asphyxia	-	+	-	+/-
**63**	8	0.2	CVI	Chromosomal deletion	+	+	-	+/-
**64**	13	-0.1	CVI	Perinatal asphyxia	-	+	-	-
**65**	9	0.1	CVI	Cerebral palsy	-	+	-	-
**66**	7	0.3	CVI	Bosch-Boonstra-Schaaf optic atrophy syndrome (BBSOAS)	+	-	-	-

Supplementary Table 2. Outcome of linear regression analyses on the data shown in Figure 2. Results of the regression analysis of the SA scores for threshold optotype sizes (top, Figure 2A), large, easy discernible optotypes (middle, Figure 2B), and the SA scores for easy discernible optotypes corrected for the reaction times in the visual detection task (VDT, bottom, Figure 2C).

**$SA_{treshold}$ ~ group + age**	**Param**	**Estimate**	**SE**	**tValue**	**pValue**
Intercept (NS) at mean age	β_0	1108	53	20.8	<0.0001
VI_o-NS	β_1	346	98	3.51	0.0006
CVI-NS	β_2	507	111	4.54	<0.0001
CVI-VI_o	β_2-β_1	161	98	1.25	0.2123
age	β_3	-69	18	-3.65	<0.0005

Adjusted R^2: 0.162; F-statistic vs. constant model: 11.4, p-value = 7.97e-07

**SA_{easy} ~ group + age**	**Param**	**Estimate**	**SE**	**tValue**	**pValue**
Intercept (NS) at mean age	β_0	715	18	38.5	<0.0001
VI_o-NS	β_1	183	36	5.13	<0.0001
CVI-NS	β_2	367	38	9.60	<0.0001
CVI-VI_o	β_2-β_1	183	45	4.05	<0.0001
age	β_3	-58	6.7	-8.76	<0.0001

Adjusted R^2: 0.519; F-statistic vs. constant model: 59.2, p-value = 9.19e-26

**SA_{easy}-VDT ~ group + age**	**Param**	**Estimate**	**SE**	**tValue**	**pValue**
Intercept (NS) at mean age	β_0	364	16	22.1	<0.0001
VI_o-NS	β_1	80	31	2.53	<0.02
CVI-NS	β_2	179	34	5.29	<0.0001
CVI-VI_o	β_2-β_1	98	40	2.46	<0.02
age	β_3	-27.67	5.9	-4.64	<0.0001

Adjusted R^2: 0.244; F-statistic vs. constant model: 17.1, p-value = 1.16e-09

Supplementary Table 3. Differences between children with CVI and children with VI_o.

**Test performed**	**CVI - VI_o**	**95% CI**	**p-Value**
SA at VA threshold: the mean reaction time to the two optotype sizes that were closest in size to the child's visual acuity	148 ms	-98 to 394 ms	0.24
SA for large optotypes: the mean reaction time to the two largest SA optotype sizes.	183 ms	94 to 273 ms	< 0.0001
VDT: the mean reaction time to a visual detection task.	85 ms	28 to 141 ms	< 0.005
ADT: the mean reaction time to an auditory detection task.	135 ms	50 to 219 ms	< 0.005
SA – VDT: mean SA reaction time to the largest SA optotype minus the mean reaction time to a visual detection task. This method adjusts SA time for the contribution of other factors, such as delays in visual stimulus detection and executing the motor response.	99 ms	19 to 178 ms	< 0.02
Delay index (DI): SA reaction time to the entire chronometric curve compared to age-matched control data.	-0.10 std	-0.89 to 0.69 std	0.81
Acuity-adjusted DI: the reaction times curves of age-matched controls were shifted to the right (towards larger optotype sizes) based on the child's visual acuity impairment.	0.77 std	-0.01 to 1.54 std	0.054

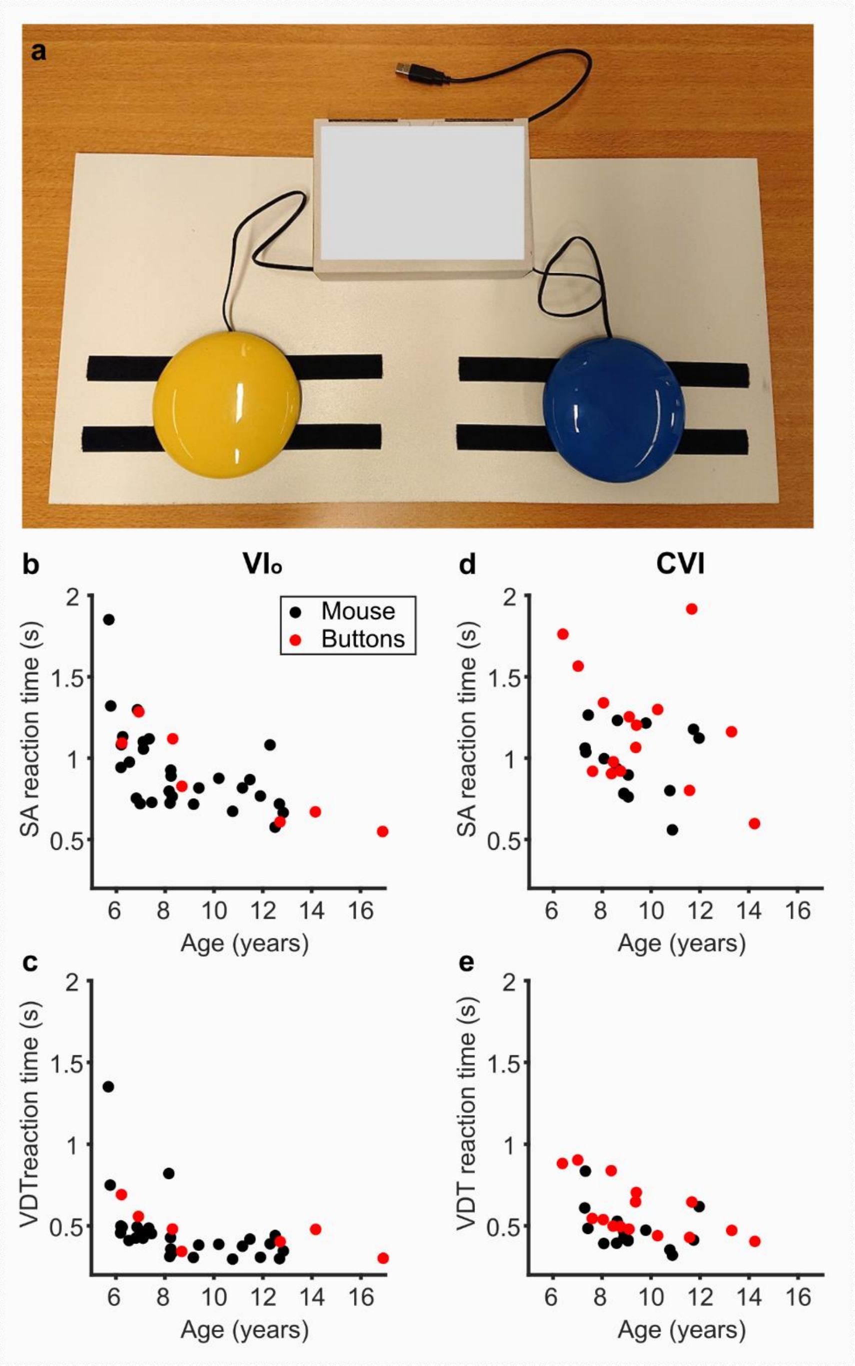

Supplementary figure 1. Clinical application of the speed acuity task
(A) A computer mouse can be difficult to operate for children with motor deficiencies. At Royal Dutch Visio, they were therefore replaced with big buttons that are applicable to a larger demographic. Picture taken by N. Tanke. **(B-C)** Mean reaction times to the two largest optotype sizes **(B)** and the VDT **(C)** for children with VI_o, using the computer mouse (black dots) or the big buttons (red dots). No differences in reaction times were found between the use of the computer mouse and the big buttons during the SA-test and the VDT (SA; 75 ms longer with buttons (95% CI: -108 to 259 ms), t(33) = 0.83, p = 0.41, VDT; 69 ms longer with buttons (95% CI: -87 to 225 ms), t(33) = 0.90, p = 0.37, linear regression). **(D-E)** Same as **(B-C),** but for the children with CVI, who reacted significantly slower with the buttons than with the mouse during both tasks (SA; 218 ms slower with buttons (95% CI: 2 to 436 ms) t(27) = 2.07, p = 0.048, VDT; 134 ms slower with buttons (95% CI: 36 to 231 ms), t(27) = 2.81, p = 0.009). Produced in MATLAB 2020b (www.mathworks.com).
